# Measuring the acute effect of insulin infusion on ATP turnover rate in human skeletal muscle using phosphorus-31 magnetic resonance saturation transfer spectroscopy

**DOI:** 10.1002/nbm.1519

**Published:** 2010-03-31

**Authors:** Ee Lin Lim, Kieren G Hollingsworth, Peter E Thelwall, Roy Taylor

**Affiliations:** a Institute of Cellular Medicine, Newcastle Magnetic Resonance Centre, Newcastle UniversityNewcastle upon Tyne, UK

**Keywords:** MRS, ATP turnover rate, insulin stimulation, time course, inorganic phosphate, insulin sensitivity

## Abstract

Mitochondrial dysfunction has been proposed to underlie the insulin resistance of type 2 diabetes. However, the relative time course of insulin action in stimulating ATP turnover rate and glucose uptake in skeletal muscle has not been examined. These two parameters were measured in young healthy subjects using the ^31^P MRS saturation transfer method in conjunction with the euglycaemic hyperinsulinaemic clamp technique respectively. Glucose infusion rate rose rapidly from 0 to 2.90 ± 0.11 mg/kg_ffm_/min during the first 10 min of insulin infusion and further to 6.17 ± 0.57 mg/kg_ffm_/min between 15 and 45 min. In contrast, baseline ATP turnover rate was 9.0 ± 0.4 µmol/g/min of muscle and did not change during the first 45 min of insulin infusion. Between 50 and 80 minutes ATP turnover rate increased by 8% and remained steady to 150 minutes (9.7 ± 0.5 µmol/g/min of muscle, *p* = 0.03 *vs* baseline). The *in vivo* time course of insulin stimulation of skeletal muscle ATP turnover rate is not consistent with a rate limiting effect upon the initiation of insulin-stimulated glycogen synthesis. Copyright © 2010 John Wiley & Sons, Ltd.

## INTRODUCTION

ATP is the universal energy currency in living cells for supporting the energy demands of various cellular activities and functions whilst insulin is the key regulator in fuel metabolism. In healthy skeletal muscle the majority of ATP production occurs in the mitochondria by oxidative phosphorylation. Mitochondrial dysfunction has been reported in skeletal muscle in type 2 diabetes (1), insulin-resistant offspring of type 2 diabetes patients (2), obesity (3) and in age-related insulin resistance (4,5), and it has been proposed that there is a causative link between the impairment of oxidative capacity and pathogenesis of type 2 diabetes in humans (6).

Using phosphorus-31 magnetic resonance spectroscopy (^31^P MRS), several research laboratories have applied magnetization saturation transfer methods to calculate unidirectional ATP synthesis rate (7–9). Using this methodology, it has been reported that insulin increases ATP turnover rate in muscle of normal subjects but not in insulin resistant subjects (10,11). This has led to a widespread acceptance that a defect in ATP generation could be a direct cause of insulin resistance (2,11–14).

However, all previous studies measured ATP turnover rate in muscle after an insulin infusion of 2–8 h duration whereas insulin is a rapidly acting regulator of metabolism which brings about major metabolic changes within minutes. The time course of stimulation of ATP turnover rate during the rapid onset of insulin's action on metabolic processes has not been previously examined. We have applied the ^31^P MRS saturation transfer method continuously during the first 150 min of insulin stimulation in normal subjects to examine whether the time course of ATP stimulation is consistent with a primary role in insulin's metabolic effects.

## MATERIALS AND METHODOLOGY

### Subjects

Seven healthy volunteers (3 male, 4 female) without family history of diabetes were studied. None were glucose intolerant or taking any medication known to affect glucose tolerance or insulin sensitivity (for example steroids, beta-blockers or diuretics). Anthropometric and metabolic characteristics of the subjects are summarised in Table 1. All participants refrained from any physical exercise during the 3 days preceding the studies and fasted overnight for 12 h before the experiments. The study protocol was approved by the Newcastle upon Tyne Research Ethics Committee 2, and informed consent was obtained from all subjects.

**Table 1:** Clinical characteristics of study subjects. Values are means ± SE

Male : female	3 : 4
Age (years)	28 ± 2
Weight (kg)	66.6 ± 6.6
Body Mass Index (kg/m^2^)	22.9 ± 1.7
Fat mass (kg)	14.9 ± 1.4
Fat free mass (kg)	51.7 ± 5.4
Estimated body fat (%)	22.6 ±1.1
Fasting blood glucose (mmol/l)	4.6 ± 0.1
Fasting plasma insulin (pmol/l)	49 ± 6
HbA_1C_ (%)	5.3 ± 0.2

### ^31^P MRS examinations

Magnetic resonance data were acquired using a 3T Achieva scanner (Philips, Best, The Netherlands) with an in-built body coil used for imaging and a 14-cm diameter surface coil for phosphorus spectroscopy. Subjects remained supine inside the magnetic resonance spectrometer with the phosphorus coil positioned beneath the left calf during each investigation. To prevent movement during each study, the coil was secured in place using fabric straps around the calf. All seven subjects were studied twice on identical study days and the fourteen sets of ATP turnover rate measurements obtained were averaged.

### ATP turnover rate measurements

**Figure 1:**
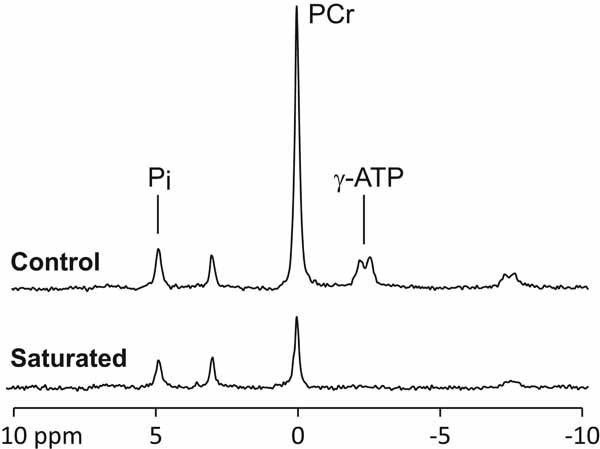
Patient spectra from the saturation transfer measurement showing (bottom) the saturation of γ-ATP at -2.38 ppm and (top) the control saturation at (2.38 ppm). The differences in the amplitudes of PCr and *P*_i_ show the saturation transfer effect.

**Figure 2:**
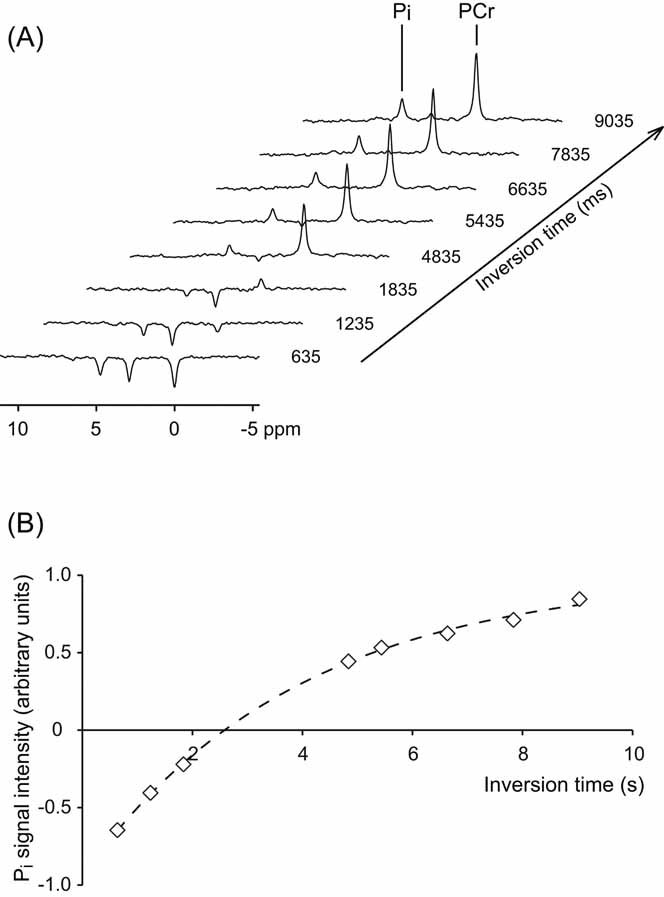
(A) Inversion recovery data showing *P*i, PDE and PCr during inversion recovery and saturation. 12Hz line broadening has been applied though fitting is in time domain. (B) Inversion recovery curve for *P*_i_ plotted through the eight inversion recovery data points. In this case, *T*_1_* = 3.90s.

Resting ATP turnover was measured using saturation transfer sequence between γ-ATP and *P*_i_ (7). For each measurement, the *P*_i_ concentration was estimated from a 1D-ISIS spectrum centred on the gastrocnemius and soleus muscles (TR = 25 s, 8 averages, duration 3.3 min). Broadband decoupling was applied to data acquisition in all experiments: the γ-ATP resonances were assumed to have concentration 8.2 mM in line with previous studies (15,16): the resonance frequency was placed mid-way between the γ-ATP and *P*_i_ resonances to ensure as even an excitation as possible at 3T. The pseudo-first order rate constant of the ATP synthase reaction was then estimated by a saturation transfer experiment as follows. The steady state-magnetization of *P*_i_ was measured during selective irradiation of γ-ATP (*M*_z_) and compared with the equilibrium *P*_i_ magnetization with the irradiation placed symmetrically down-field from the *P*_i_ frequency (TR = 25 s, bandwith = 3,000 Hz, 2,048 points, 16 averages each, duration 13.3 min) (*M*_O_). The resonant frequency was set at the *P*_i_ frequency to allow symmetrical saturation pulses about that resonance. The fractional reduction of *P*_i_ magnetization upon saturation of γ-ATP, (*M*_O_–*M*_z_)/ *M*_O_, was used to calculate the pseudo-first order rate constant using the Forsen-Hoffman equation: *k*_1_ = [(*M*_O_–*M*_z_)/ *M*_O_](1/*T*_1_*), where *T*_1_* is the spin-lattice relaxation time for the phosphorus nucleus of *P*_i_ when ATP is saturated (17). *T*_1_* was measured using an inversion recovery experiment (τ_1_–180°–τ_2_–90°–acquire, TR = 25 s, 4 averages, duration 13.3 min), while saturation of γ-ATP was performed during the delay times τ_1_ and τ_2_. Broad-band proton decoupling and 1D ISIS localisation was used. Eight variable τ_2_ time delays were used ranging from 635–9,035 ms. Therefore the time required for a complete saturation transfer measurement was 26.7 min. Unidirectional turnover rate of ATP synthesis was then calculated by multiplying the constant *k*_1_ by the *P*_i_ concentration. Analysis of all spectra was performed with jMRUI (version 3.0) (18) using AMARES (19). Prior knowledge was used to solve the datasets as follows. Single Lorentzian peaks were modelled for inorganic phosphate (*P*_i_), phosphodiesters (PDE) and phosphocreatine (PCr). The α- and γ-ATP resonances were modelled as Lorentzian duplets of equal magnitude separated by 18Hz. Due to the bandwidth of the pulses used and tuning *P*_i_ to be on resonance, the β-ATP resonance is only weakly detected and is not modelled. To fit the saturation transfer and inversion recovery experiments the zero and first order phasing and the *P*_i_ linewidth was determined from the control saturation scan. Phase values were then applied using the previous prior knowledge to fit the saturation, control and inversion recovery data. This provides sufficient stability to fit inversion recovery spectra with peaks of different *T*_1_* value using the AMARES algorithm. Sample spectra are given in Figures 1 and 2. In preparatory work for the first study using this implementation of the saturation transfer technique (9), three healthy, fasting subjects were studied three times on one day without repositioning of the r.f. coil yielding an intra-day variability of 6.5% in ATP turnover rate, and were studied twice more on two further separate days to examine the effect of coil repositioning, yielding an interday reproducibility of 8% in ATP turnover rate.

Two consecutive baseline measurements were acquired from 0830 to 0930 (−60 to −30 min and -30 to 0 min respectively relative to the start of infusion) and during the 150 min of the hyperinsulinaemic glucose clamp, four contiguous measurements were performed (15–45 min, 50–80 min, 85–115 min, and 120–150 min). Owing to the fact that it takes 27 minutes to acquire data to measure the pseudo-first order rate constant, *k*_1_, and that *P*_i_ concentration can increase across this time, it was decided to take the average of two measurements of *P*_i_ concentration before and after the saturation transfer measurement of *k*_1_ in every case for calculations of ATP turnover rate.

### Glucose clamp studies

For all experiments, participants travelled to the magnetic resonance facility by taxi and were transported within the centre by wheelchair. At 0800 (−90 min), one cannula was inserted into a large antecubital vein for administration of intravenous glucose and insulin. A second cannula was inserted into the contralateral wrist vein for the purpose of blood sampling. Use of a hand warming device ensured arterialisation of venous blood. Conditions of fasting glycaemia and standardised hyperinsulinaemia were achieved for 150 min using the euglycaemic hyperinsulinaemic clamp technique (20). Insulin (Actrapid; NovoNordisk, Bagsvaerd, Denmark) was administered from 0930 to 1200 (0–150 min) as a primed-continuous infusion (primed followed by 40 mU/m^2^/min). The fasting blood glucose concentration for each subject was maintained by adjusting the infusion rate of a 20% dextrose solution based on blood glucose measurements performed at 5 min intervals. Whole body insulin sensitivity was determined from calculated whole body glucose disposal during the last 30 min of the hyperinsulinaemic glucose clamp (20). Glucose infusion rate was assumed to represent whole body glucose disposal since endogenous glucose release is negligible at this level and duration of hyperinsulinaemia in normal subjects (21).

### Blood glucose and plasma insulin

Blood glucose concentration was measured by the glucose oxidase method (YSI glucose analyser; YSI, Yellow Springs, OH). Plasma insulin concentration was measured using an enzyme-linked immunosorbent assay kit (DAKO, Ely, UK). Blood glycosylated haemoglobin (HbA_1C_) level was measured as an indicator of average blood glucose control in the preceding 8 weeks using high performance liquid chromatography (TOSOH, Tokyo, Japan).

### Statistical analysis

Statistical analyses were performed using SPSS 15.0 software (SPSS Inc., Chicago, USA). Data are presented as means ± SE in the text and in the figures, unless otherwise stated. Prior to any comparisons, distribution was tested for normality using the Shapiro-Wilk W-test. For our study of seven subjects, non-parametric Wilcoxon test was used to determined within group differences (22). Statistical significance was accepted at *p* < 0.05.

## RESULTS

### Whole body glucose metabolism

**Figure 3:**
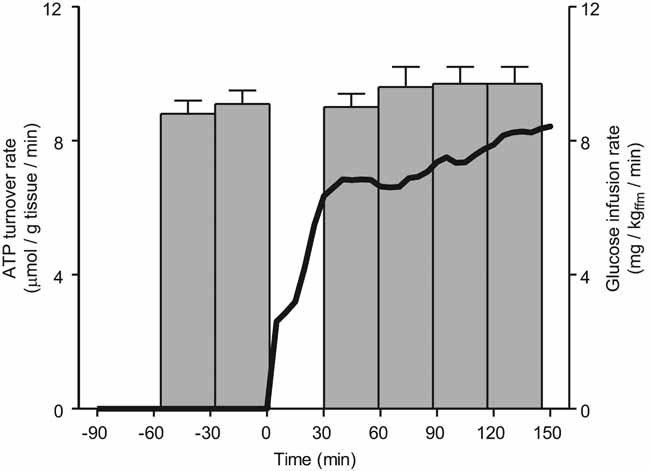
Simultaneous time course change in ATP turnover rate (represented in histogram bars) and glucose infusion rate (solid line) during the euglycaemic hyperinsulinaemic clamp.

Mean blood glucose concentration during baseline measurement was 4.6 ± 0.1 mmol/l and was maintained at this concentration during the euglycaemic hyperinsulinaemic period (4.7 ± 0.1 *vs* 4.6 ± 0.1 mmol/l). Fasting plasma insulin concentration was 49 ± 6 pmol/l, and this increased approximately 10-fold within 15 min of the clamp. Plasma insulin concentration remained constant for the duration of the clamp (456 ± 19 pmol/l, *p* < 0.001 *vs* baseline). A rapid increase in whole body glucose disposal rate was observed via a rise of glucose infusion rate from 0 to 2.90 ± 0.11 mg/kg_ffm_/min during the first 10 min and further to 6.17 ± 0.57 mg/kg_ffm_/min between 15–45 min (Fig. 3). There was a subsequent slower increase to 8.31 ± 0.67 mg/kg_ffm_/min during the 120–150 min period.

### Resting ATP turnover rate

Mean baseline ATP turnover rate was 9.0 ± 0.4 µmol/g/min of muscle. During the first 45 min of the clamp, there was no increase in ATP turnover rate to match the acute onset of the metabolic actions of insulin reflected by the steep increase in glucose utilisation. Between 50 and 80 min after the insulin infusion was commenced, ATP turnover rate increased by 8% and remained steady until the end of the clamp (9.7 ± 0.5 µmol/g/min of muscle, *p* = 0.03 *vs* baseline).

The average of the two *P*_i_ concentration acquired at baseline was 3.62 ± 0.07 mmol/l. *P*_i_ concentration did not change during the early period of insulin infusion (15–45 min period: 3.58 ± 0.08 mmol/l, *p* = 0.463). Thereafter, *P*_i_ concentration increased to 3.98 ± 0.09 mmol/l between 50 and 80 min (*p* < 0.05 *vs* baseline). This corresponded to a 14% increase compared to baseline (Table 2).

**Table 2:** Components of the ATP turnover rate measurement at baseline and during insulin stimulation. Values are means ± SE

		During Isoglycaemic hyperinsulinaemic clamp			
		
	Baseline	15–45 min	50–80 min	85–115 min	120–150 min
*k*_1_ (s^−1^)	4.09 ± 0.18	3.89 ± 0.20	3.91 ± 0.27	4.01 ± 0.26	4.08 ± 0.29
*P*_i_ (mmol/l)	3.62 ± 0.07	3.58 ± 0.08	3.98 ± 0.09	3.99 ± 0.13	3.90 ± 0.11
*T*_1_* (s)	4.00 ± 0.10	4.30 ± 0.12	4.10 ± 0.15	4.25 ± 0.15	4.08 ± 0.12
M_z_/M_0_ (-)	0.73 ± 0.01	0.72 ± 0.01	0.73 ± 0.01	0.72 ± 0.01	0.73 ± 0.01

There was no significant change in *k*_1_ for ATP turnover rate between baseline and insulin-stimulated conditions (Table 2). This was reflected by there being no significant change in either the *T*_1_* of *P*_i_ (the longitudinal relaxation time during γ-ATP saturation) or the fraction of *P*_i_ signal during saturation compared to control saturation (M_z_/M_0_) under insulin stimulation. As *k*_1_ was unchanged, all change in ATP turnover rate was derived from change in *P*_i_ concentration.

## DISCUSSION

To our knowledge, this is the first study to investigate the acute effect of insulin on muscle ATP turnover rate, and to track the time course of ATP turnover rate during physiological hyperinsulinaemia. Insulin is responsible for increasing whole body glucose disposal mainly by stimulating glycogen synthesis in the transition from fasted to fed state (23). Carbon-13 magnetic resonance spectroscopy studies have shown physiological hyperinsulinaemia to increase muscle glycogen concentration in as little as 15 min in healthy individuals (24,25), which is reflected in the rapid increase in glucose disposal rate observed in our experiments.

The glucose infusion rate data shown in Figure 3, illustrate that the highest rate of change in glucose disposal occurs in the first 30–60 min of euglycaemic hyperinsulinaemia. The ATP turnover rate was unchanged during the first 20–50 min of the clamp, with an 8% increase after 50 min. There are notable differences with other reported measurements of ATP turnover rate during physiological hyperinsulinaemia. The single most important difference lies in the timing of the measurements. The original report of mitochondrial dysfunction associated with diabetes measured ATP turnover rate averaged over 30–150 min of euglycaemic hyperinsulinaemia (10) while subsequent studies compared the later effects (120–350 min) of insulin stimulation of ATP turnover rate (8,11). The long scan duration limited the time resolution and prevented examination of time course. However, in order to determine the relationship between parameters it is important to measure ATP turnover rate during the onset of insulin action, as early as 15 min of insulin stimulation (24). Other processes, separate from insulin's effect on muscle glycogen synthesis, such as insulin's effect on mitochondrial fusion and proliferation (26,27) and mitochondrial protein synthesis (28), may affect ATP turnover rate on a timescale of 4–8 h of insulin stimulation. These processes are temporally dissociated from and not relevant to the early metabolic effects of insulin. They are likely to affect data on ATP turnover rates in the longer studies of prolonged hyperinsulinaemia.

In the present study insulin stimulation for 150 min brought about an 8% increase in ATP turnover rate, less than the 11–90% previously observed in normal control subjects in the literature (2,11). The report of a 90% increase in ATP turnover rate in insulin-sensitive subjects with insulin stimulation (2) related to extreme phenotypes of insulin sensitivity. Additionally, the insulin resistant offspring were reported to have a low basal resting ATP turnover rate. This has not been confirmed in subsequent studies comparing subjects with and without type 2 diabetes when subjects have been matched for habitual physical activity (9,11). During a 240 min period of hyperinsulinaemia, Szendroedi *et al.* reported a 10.5% increase in ATP turnover rates in the diabetic subjects, 10.6% in the age matched controls, and a 26% increase in ATP turnover rate in the young controls (11). It has been widely postulated within the MR and diabetes communities that defects in mitochondrial function may underlie the reduced glucose disposal rate during hyperinsulinaemia in type 2 diabetes (11–14). Although the data suggest a relationship between insulin sensitivity and ATP turnover rate, this does not indicate cause and effect. Hence, the extrapolation of hypothesis from the original observations (2) to the pathophysiology of type 2 diabetes itself has not been well justified.

In addition to the shorter period of insulin stimulation examined in the present study as explanation for observing a lesser magnitude of increase of ATP turnover rate with insulin, other factors such as surface coil placement, coil diameter and acquisition sequences could affect the results in comparing between studies. The relative composition of the r.f. coil's sensitive volume of gastrocnemius and soleus muscles requires consideration as soleus muscle, which has predominantly Type I muscle fibres, has been shown to have higher resting ATP turnover rate than gastrocnemius (29). We used a larger coil than used by Szendroedi *et al.* (11) (14 cm diameter compared to 10 cm) and thus would expect to have a higher percentage of contribution of signal from the soleus muscle.

ATP turnover rate is calculated from both the intramyocellular *P*_i_ concentration and the measured rate constant *k*_1_. To ensure our estimates of ATP turnover rate were as accurate as possible during the rapidly changing *P*_i_ concentration in the first hour after insulin stimulation, the average of the *P*_i_ concentrations before and after the 27-min saturation transfer measurement was used. We observed insulin-stimulated *P*_i_ concentration to increase by 14% until the end of the euglycaemic hyperinsulinaemic clamp, in agreement with previous studies (11,30). The concentration of *P*_i_ in the muscle tissue may be an important mechanism by which insulin regulates ATP synthesis (31): there was no change in measured reaction rate, *k*_1_ across the two hour duration of this clamp, or in any of the parameters measured to calculate *k*_1_. Such changes were implied by the early work in this field on young insulin sensitive volunteers (2,10).

Measurements of ATP turnover rate by ^31^P saturation transfer method report flux of ATP through *P*_i_ incorporation into ATP, and are thus comprised of ATP generated by glyceraldehyde-3-phosphate dehydrogenase and 3-phosphoglycerate kinase reactions as well as by ATP synthase. This could result in a mismatch between oxygen consumption and ATP synthesis (32), as demonstrated in studies performed in aerobic yeast suspensions *Saccharomyces cerevisiae* (32) and perfused rat myocardium (33). However, arterio-venous balance studies across the leg have shown that there is no net export of lactate from human muscle following insulinisation in either type 2 diabetic subjects or leaner normal controls such as used in the present study (34). This net measurement does not exclude the possibility of a change in redundant cycling of 3-carbon compounds through the glycolytic exchange flux, but the extent to which this cycling contributes to the observed rate of overall ATP turnover still remains to be determined (35).

A hallmark of muscle physiology is the ability to support a high energy demand for muscle function by phosphocreatine buffering of ATP, and by aerobic and anaerobic respiration. Thus postulation that muscle glycogen synthesis may be limited by ATP production, especially in resting muscle where ATP capacity is high, seems at odds with muscle function. In support of this, more recent reconsideration of the data supporting mitochondrial dysfunction as pathogenic for type 2 diabetes has concluded that the cause-and-effect relationship between mitochondrial dysfunction and insulin resistance is not supported by all data (9,36,37).

For the first time, the early time course of skeletal muscle ATP turnover in response to insulin has been quantified in young, healthy controls. There was no increase in ATP turnover rate during the first hour of administering a high physiological concentration of insulin, the timescale where glycogen synthesis is initiated. ATP turnover rate was observed to increase only after 1 h, indicating that glycogen synthesis is not limited by ATP availability in healthy controls, with implications for hypotheses about the origins of defective insulin sensitivity in type 2 diabetes.

## Abbreviations used

^31^P MRS: phosphorus-31 magnetic resonance spectroscopy
^13^C MRS: carbon-13 magnetic resonance spectroscopy
ffm: fat free mass
